# Mesenchymal Stromal Cell Therapy in Cardiovascular Disease: Reconsidering Clinical Inconsistency Through the Mechanism–Endpoint Relationship

**DOI:** 10.31083/RCM52521

**Published:** 2026-08-20

**Authors:** Luca Bonanni, Nicola Ferri

**Affiliations:** ^1^Department of Medicine, Ospedale dell’Angelo, 30174 Venice, Italy; ^2^Department of Medicine, University of Padua, 35128 Padova, Italy

**Keywords:** mesenchymal stromal cells, myocardial infarction, heart failure, paracrine communication, extracellular vesicles, immunomodulation, clinical trials as topic, translational research, endpoint determination, regenerative medicine

## Abstract

Mesenchymal stromal cell (MSC) therapy has been investigated for more than two decades as a regenerative approach in cardiovascular disease. Preclinical studies have shown reproducible biological activity; however, clinical translation has been less convincing. Trials in acute myocardial infarction (AMI) and heart failure (HF) have established feasibility and an acceptable safety profile; however, effects on functional and imaging parameters remain modest, and a consistent impact on mortality, hospitalization, and other hard clinical outcomes has not been demonstrated.

## 1. Introduction

Mesenchymal stromal cells (MSCs) have been studied in regenerative cardiovascular medicine because they are relatively accessible from several adult and perinatal sources, can be expanded *ex vivo*, and display a broad range of biological activities relevant to tissue repair. Early experimental work raised the possibility that MSCs might contribute to myocardial regeneration through direct differentiation into cardiomyocyte-like cells and vascular structures [1,2]. That interpretation guided much of the early clinical development.

The original differentiation hypothesis has progressively weakened. Lineage-tracing studies and serial *in vivo* imaging have shown that durable engraftment is uncommon and that few administered cells remain within the myocardium beyond the first weeks after delivery [1,3,4]. Direct evidence of functional cardiomyocyte differentiation has not been robustly confirmed. Current evidence indicates that the effects of MSCs are mediated mainly by paracrine signalling and immunomodulatory activity rather than by tissue replacement [1,2,5,6].

Paracrine signalling refers, in this context, to the release of bioactive factors—growth factors, cytokines, chemokines, and extracellular vesicles—that act on neighbouring cells to influence inflammation, angiogenesis, fibroblast behaviour, and cardiomyocyte survival. Immunomodulation describes the capacity of MSCs to alter the activity of innate and adaptive immune cells, including macrophage polarisation, regulatory T-cell induction, and dampening of pro-inflammatory cascades [5,7]. Both processes depend on the presence of viable, metabolically competent cells, although emerging evidence suggests that nonviable cellular material may also engage host repair mechanisms [8].

Despite a coherent biological rationale, clinical translation has been less consistent. Trials in cardiovascular disease have shown feasibility and acceptable safety, but improvements in functional parameters such as left ventricular ejection fraction (LVEF), scar size, or ventricular volumes remain modest and variable [2,9,10,11]. Effects on major clinical outcomes have not been consistently demonstrated. Several factors plausibly contribute: low myocardial retention after delivery, short cellular persistence, marked heterogeneity in MSC products, differences in disease stage and patient biology, and methodological variability across trials [9,12,13,14]. Each of these has been examined in previous reviews.

These explanations may be incomplete. They tend to treat MSC therapy as a regenerative intervention whose efficacy is reduced by technical or biological inefficiency. They do not directly address whether the expected effects have been assessed through endpoints adequately matched to the biology being tested. If MSC activity depends mainly on transient paracrine and immunomodulatory signals, its expected biological footprint is modulatory rather than regenerative in the strict sense. A short-lived intervention may shape early inflammation, microvascular repair, and immune polarisation without necessarily producing durable structural change. Whether endpoints traditionally selected in cardiovascular trials—long-term LVEF, scar size, mortality—are optimally sensitive to this type of signal is a question that has received comparatively little attention.

This review does not aim to provide another general summary of MSC trials in cardiovascular disease. It interprets the clinical inconsistency of MSC therapy through the relationship between biological exposure, disease context, and endpoint selection. Within this framework, mechanisms beyond classical paracrine signalling are considered, the determinants of effective biological exposure are integrated, and the implications for clinical study design are discussed. The novelty is not the recognition that MSC trials are heterogeneous. It lies in the proposal that part of the heterogeneity may be a measurement artefact, arising from the gap between transient modulatory biology and trial architecture calibrated to detect durable change.

## 2. Literature Selection

This narrative review is based on a structured but non-systematic search of PubMed/MEDLINE, Scopus, and Web of Science. The search covered the period from January 2000 to October 2025 and combined the terms “mesenchymal stem cells”, “mesenchymal stromal cells”, “MSC”, “cell therapy”, “acute myocardial infarction”, “heart failure”, “ischemic cardiomyopathy”, “cardiac regeneration”, “paracrine signalling”, “immunomodulation”, “extracellular vesicles”, “exosomes”, “retention”, “engraftment”, “biodistribution”, “clinical trial”, “meta-analysis”, and “endpoint”. Reference lists of retrieved articles were screened for additional sources.

Priority was given to landmark randomised clinical trials, systematic reviews and meta-analyses, mechanistic primary studies, and recent high-quality review articles. Pre-clinical mechanistic work was included when directly relevant to a mechanism discussed in human studies. Studies not focused on cardiovascular translation were excluded, except where they provided foundational evidence on MSC biology, manufacturing variability, or biodistribution. No formal risk-of-bias assessment was performed, and the review is not registered as a systematic review.

## 3. From Regenerative Replacement to Modulatory Biology

MSCs were introduced into cardiovascular medicine within an explicitly regenerative framework. Early *in vitro* and small-animal experiments suggested that bone marrow–derived MSCs could acquire cardiomyocyte markers under specific culture conditions, and a few *in vivo* studies appeared to support myogenic conversion after intramyocardial injection [1,2,3]. The clinical concept that followed was straightforward: deliver cells to ischaemic myocardium, allow them to engraft, and replace lost contractile tissue.

Subsequent work has progressively undermined this interpretation. Lineage-tracing experiments did not confirm appreciable cardiomyocyte differentiation of administered MSCs. Serial bioluminescence imaging and biodistribution studies showed rapid loss of administered cells within days to weeks, regardless of route [3,4]. Even in studies reporting functional improvement, the magnitude of cell engraftment appeared too small to account for the observed effect through tissue replacement [1,3,6]. The biological mechanism behind any benefit had to be sought elsewhere.

Attention shifted toward paracrine signalling. MSCs release a complex secretome that includes vascular endothelial growth factor (VEGF), hepatocyte growth factor (HGF), insulin-like growth factor-1 (IGF-1), stromal cell-derived factor-1α (SDF-1α/CXCL12), and several anti-apoptotic and anti-fibrotic mediators [6]. Some of these factors act in a coordinated manner; VEGF and SDF-1α have shown synergistic effects on endothelial progenitor cell migration, proliferation, and differentiation in co-culture systems with vascular smooth muscle cells [15]. Immunomodulatory activity has been characterised in detail, including suppression of T-cell proliferation, polarisation of macrophages towards a reparative phenotype, induction of regulatory T cells, and reduction of neutrophil-driven tissue injury [5,7]. These activities provide a more parsimonious explanation for the modest, time-limited improvements observed in trials than direct tissue replacement does.

A historical reassessment is helpful here. Cardiac cell therapy emerged as a clinical field roughly twenty-five years ago, and its trajectory has been shaped by repeated cycles of expectation and revision, as recently summarised by Zhang and colleagues [16] in a comprehensive overview of the past two decades of cell- and cell-product-based therapies for heart failure (HF). The shift from a regenerative to a modulatory paradigm is one of the most consequential of these revisions. It carries an implicit consequence for trial design that has not always been recognised: a modulatory therapy is unlikely to produce the same kind of clinical signal as a regenerative one and is unlikely to be detectable by the same endpoints.

## 4. Mechanisms Beyond Simple Paracrine Signalling

Restricting MSC activity to a generic paracrine model is likely an oversimplification. Several mechanisms operate concurrently, and their relative weight may vary with disease context, route of delivery, and product characteristics [17].

**Secretome and growth factor signalling. **MSCs secrete a complex mixture of soluble mediators that influence angiogenesis, cardiomyocyte survival, and fibroblast behaviour. VEGF and SDF-1α appear particularly relevant for revascularisation; *in vitro* work has shown that their combined action increases tube formation, migration, and proliferation of endothelial progenitor cells, while their effect on vascular smooth muscle cells is more variable [15]. HGF and IGF-1 contribute to cardiomyocyte cytoprotection and limitation of apoptosis [6]. The secretome composition is influenced by donor factors, culture conditions, and preconditioning.

**Immunomodulation. **MSCs interact with innate and adaptive immune compartments through cell–cell contact and soluble factors, including indoleamine 2,3-dioxygenase (IDO), transforming growth factor beta (TGF-β), prostaglandin E2, and interleukin-10. The result is a shift in the local cytokine environment, suppression of pro-inflammatory cascades, and polarisation of macrophages towards a reparative phenotype [5,7]. In acute myocardial injury, this activity may modify the temporal pattern of inflammation and microvascular dysfunction in a way that downstream remodelling cannot easily reveal.

**Activity independent of cell viability. **A particularly relevant observation comes from work by Vagnozzi and colleagues [8], who showed in murine ischaemia–reperfusion that the functional benefit of intracardiac stem cell injection is not associated with the production of new cardiomyocytes. Intramyocardial injection of two distinct adult stem cell types, of cells killed by freezing and thawing, or of a chemical inducer of the innate immune response all elicited a similar regional accumulation of CCR2+ and CX3CR1+ macrophages, altered cardiac fibroblast activity, reduced extracellular matrix content in the border zone, and improved the mechanical properties of the injured area [8]. The implication is striking: at least part of the observed biological effect may not require sustained MSC paracrine activity. It may instead reflect a stimulus delivered to the host immune system. This finding does not invalidate paracrine mechanisms; it suggests that the host response itself is part of the therapeutic chain.

**The reservoir effect. **After intravenous delivery, a large fraction of MSCs is trapped in pulmonary microvasculature; smaller fractions distribute to liver, spleen, and bone marrow [4,13]. Cells retained outside the heart may continue to exert systemic immunomodulatory effects through soluble mediators or extracellular vesicles, potentially shaping the inflammatory milieu seen by the myocardium. This “reservoir” model is supported by experimental work showing that intravenously infused human MSCs, embolised in mouse lung, are activated to secrete the anti-inflammatory protein TSG-6, with downstream reduction of inflammatory responses, reduced infarct size, and improved cardiac function after myocardial infarction—an effect lost when MSCs are transduced with TSG-6 small interfering RNA (siRNA) [18].

**Extracellular vesicles. **MSC-derived extracellular vesicles (EVs), including exosomes, transport proteins, lipids, and regulatory RNAs to recipient cells. They exhibit immunomodulatory effects, including suppression of pro-inflammatory cytokines, promotion of regulatory T cells, and shaping of anti-inflammatory microenvironments [19,20]. In preclinical models of ischaemia–reperfusion injury, they reproduce several of the effects attributed to whole-cell therapy [19]. EVs represent a related but distinct therapeutic direction: a cell-free product that may be more amenable to standardisation and quality control than living cells, although questions of dose, biodistribution, and long-term safety remain open [20].

**Hemostatic and immunothrombotic interactions. **A further dimension of the host response concerns the interaction of MSCs with coagulation. MSCs can promote thrombin generation through tissue factor expression and phosphatidylserine exposure, while also engaging complement, platelets, and innate immune pathways; counter-regulatory mechanisms, including adenosine-mediated platelet inhibition and immune reprogramming after cellular clearance, contribute to a context-dependent effect [21]. Major thrombotic events appear uncommon in clinical practice, although subclinical activation of coagulation may occur, and functional assays—rather than tissue factor expression alone—appear necessary to estimate effective procoagulant potential [21]. This interaction is most relevant to the safety profile and the host inflammatory status that conditions MSC activity.

These mechanisms are not mutually exclusive. Secretome activity, immunomodulation, cell-debris–driven innate responses, systemic effects from extracardiac reservoirs, and EV-mediated signalling may operate in parallel, with relative contributions shifting according to the route of delivery, disease stage, and product characteristics. The practical consequence is that a single trial endpoint is unlikely to capture the full range of biological activity, and the absence of a regenerative signal does not imply biological inactivity.

## 5. Delivery, Retention, Persistence, and Biological Exposure

Therapeutic activity of cell-based products depends on the presence of viable, secretorily competent cells within or near the target tissue. For MSCs in cardiovascular disease, the effective biological exposure of the myocardium is governed by three interrelated variables: how many cells reach the target site, how long they remain there, and how much of their secretory activity occurs during that window. All three are currently limiting.

**Routes of administration. **Three main routes have been used in clinical studies: intravenous (peripheral or central), intracoronary, and intramyocardial (transendocardial or transepicardial). Intravenous delivery is the simplest and least invasive but is associated with marked pulmonary trapping. Intracoronary infusion increases first-pass exposure of the myocardium but is constrained by epicardial vessel anatomy and risk of microvascular obstruction. Intramyocardial injection delivers cells directly into the wall of the ventricle, including chronically infarcted regions where coronary flow is reduced, and tends to achieve the highest initial myocardial cell densities [13,17]. Reported cell doses in adult cardiovascular trials have spanned a broad range, typically from about 10^6^ to several × 10^8^ cells, with substantial variation across trials [2,9,22,23]; the POSEIDON, TRIDENT, TAC-HFT, and MSC-HF trials, for instance, used doses between approximately 20 and 200 million cells [24,25,26,27].

**Initial retention. **Radiolabelled tracking studies have shown that only a small fraction of cells delivered to the heart remain at the target site shortly after administration. Reported myocardial retention values are highly variable but generally low; estimates in the order of a few per cent of the administered dose at one hour after intracoronary infusion have been reported, with intramyocardial delivery yielding somewhat higher early retention [13,17]. The remainder of the dose is rapidly redistributed to non-target organs, predominantly the lungs after intravenous infusion and lungs, liver, and spleen after coronary routes [4,13]. Recent comparative analyses across routes, including intracoronary, intravenous, retrograde coronary venous, intramyocardial, and intrapericardial delivery, conclude that low retention is a common feature shared by all available approaches and a major determinant of efficacy in animal models and human trials [17].

**Persistence. **Even when initial retention is comparatively favourable, persistence is short. Bioluminescence imaging and positron emission tomography (PET) studies in animals have shown rapid decline of viable MSCs within days, with most studies reporting near-complete clearance over the first few weeks [3,4]. In humans, indirect evidence is consistent with similar kinetics. Long-term engraftment of administered MSCs in the human heart has not been convincingly demonstrated.

**Implications for biological exposure. **Low retention and short persistence converge on a single conclusion. The cumulative paracrine and immunomodulatory dose delivered to the myocardium is small and brief. Comparative analyses across delivery routes have proposed that improving retention is a necessary condition for translating cell-based therapy into reproducible clinical benefit, since strategies that increase the duration of cell residency tend to enhance paracrine signalling to surrounding damaged tissue [17]. Retention should therefore be regarded not as a technical detail but as a central determinant of effect size.

Persistent biological effect after rapid cell loss is not, however, biologically implausible. As discussed in the previous section, contributions from cell debris, host immune engagement, and systemic reservoirs may extend the effective therapeutic window beyond the period of measurable myocardial cell viability [8,18]. The relationship between retention and outcome is therefore real but not strictly linear, and improving retention is one of several routes to improving exposure.

## 6. Product, Donor, and Host Variability

Sources of variability in MSC therapy can be ordered by the level at which they operate: the cells themselves, the donor of origin, the manufacturing process, and the host environment. This hierarchy is useful because mitigation strategies differ at each level.

**Cell and tissue source. **Bone marrow has been the historically dominant source, partly because of earlier clinical experience. Adipose tissue and perinatal sources—umbilical cord, Wharton’s jelly, placenta—have attracted interest because of higher proliferative capacity and, in some preparations, distinct immunomodulatory profiles [2,28,29,30]. The minimal phenotypic criteria proposed by the International Society for Cell and Gene Therapy (ISCT) (plastic adherence; expression of CD73, CD90, CD105; absence of haematopoietic markers; tri-lineage differentiation) confirm broad cellular identity but do not predict *in vivo* behaviour or secretory competence [28,31,32]. Two MSC preparations labelled identically may differ substantially in potency.

**Autologous versus allogeneic preparations. **Autologous MSCs reflect the biological state of the patient, including comorbidities, age, and metabolic burden; cells obtained from older patients with diabetes or established cardiovascular disease may have reduced function compared with cells from younger healthy donors. Allogeneic MSCs allow the use of pre-screened, well-characterised donors and enable off-the-shelf availability, at the cost of potential immune recognition. In the POSEIDON trial, which directly compared autologous and allogeneic bone marrow MSCs delivered transendocardially in 30 patients with ischaemic cardiomyopathy, neither preparation triggered clinically significant donor-specific alloimmune reactions, and both produced comparable reductions in early enhancement defect (a measure of infarct size) of approximately 33% relative to baseline [24].

**Manufacturing and product qualification. **Expansion conditions, passage number, cryopreservation, and thawing influence viability and secretory profile [14,28,33]. Cells exposed to suboptimal expansion or to cryopreservation stress may retain their surface marker profile while showing reduced metabolic fitness and impaired secretion. The nominal cell dose can therefore overestimate effective biological activity. Available clinical reports often provide limited information on viability at infusion, functional potency assays, or release criteria [31], which complicates comparison across trials.

**Host responsiveness. **Patients enrolled in cardiovascular MSC trials are heterogeneous with respect to age, comorbidities, ischaemia burden, residual viability, inflammation, and stage of remodelling [14,34]. MSC activity depends on the local cellular and molecular environment of the recipient tissue; a less responsive substrate may produce a smaller measurable effect for the same biological signal. Host immunity may also recognise allogeneic MSCs, accelerate clearance, or otherwise modulate cell behaviour, and the host inflammatory status may further condition both efficacy and the hemostatic response to administered cells [7,14,21,35].

From a translational perspective, the four levels of variability affect different aspects of the therapeutic chain. Cell source and manufacturing largely determine product potency. Donor characteristics affect autologous preparations in particular. Delivery determines biological exposure. Host factors determine responsiveness. Endpoint choice and trial design determine measurement. Improvements at one level cannot fully compensate for limitations at another.

## 7. Disease Context: Acute Myocardial Infarction, Chronic Heart Failure, and Beyond

The clinical effect of MSC therapy appears to depend on the biological state of the tissue in which it is delivered. Acute myocardial infarction (AMI) and chronic HF represent two contrasting environments, and the available evidence is broadly consistent with this distinction.

**Acute myocardial infarction. **Early after coronary occlusion and reperfusion, the myocardium is the site of an active and time-limited cascade of injury and repair, including cardiomyocyte death, sterile inflammation, microvascular dysfunction, and the early activation of fibroblasts and reparative immune cells. Within this biologically dynamic window, transient paracrine and immunomodulatory signals may plausibly influence the trajectory of infarct evolution. The meta-analysis by Attar and colleagues [22], which pooled 13 randomised trials of MSC transplantation after AMI including 956 patients (468 treated, 488 control), reported a weighted mean LVEF improvement of 3.78% (95% CI 2.14–5.42; *p* < 0.001) relative to control, with a more pronounced effect when cells were administered within the first week after infarction (mean difference 5.74%; 95% CI 4.30–7.18; *p* < 0.001) [22]. The efficacy of transendocardial injection and intracoronary infusion appeared broadly similar (≈4% vs ≈3.6% LVEF gain). Effects on hard clinical outcomes were not consistently demonstrated.

**Chronic heart failure and ischaemic cardiomyopathy. **Established HF is dominated by fibrosis, ventricular remodelling, and chronically altered cardiomyocyte and fibroblast biology. Tissue plasticity is reduced. Several landmark trials have explored MSCs in this setting using diverse routes and products. POSEIDON randomised 30 patients with ischaemic cardiomyopathy to autologous or allogeneic bone marrow MSCs (20, 100, or 200 million cells) delivered transendocardially; both preparations produced improvements in ventricular remodelling, with the lowest dose (20 million cells) achieving the greatest reductions in LV volumes and ejection fraction gain [24]. TAC-HFT randomised 65 patients with ischaemic cardiomyopathy and LVEF below 50% to transendocardial autologous MSCs, autologous bone marrow mononuclear cells, or placebo; MSCs reduced infarct size by 18.9% of LV mass (95% CI –30.4 to –7.4; *p* = 0.004), improved Minnesota Living With Heart Failure scores, and increased 6-minute walking distance, while LVEF and ventricular volumes did not change significantly [25]. The MSC-HF trial randomised 60 patients with severe ischaemic HF (LVEF <45%, New York Heart Association (NYHA) II–III) 2:1 to intramyocardial autologous bone marrow MSCs or placebo; at 6 months, the primary endpoint of LV end-systolic volume was reduced by 7.6 mL in the MSC arm and increased by 5.4 mL in the placebo arm (between-group difference 13.0 mL; *p* = 0.001), with concomitant LVEF improvement of approximately 6.2 percentage points relative to placebo [26]. The TRIDENT dose-comparison study (30 patients) showed that both 20 and 100 million allogeneic MSCs reduced scar size to a similar extent, but only the higher dose produced a significant LVEF gain of 3.7 percentage points [27]. CHART-1, which enrolled patients with symptomatic ischaemic HF and randomised 315 patients (157 cardiopoietic cells, 158 sham; 271 actually treated) to MSC-derived cardiopoietic cells delivered through a retention-enhanced catheter, did not meet its primary hierarchical composite endpoint at 39 weeks; exploratory analyses suggested benefit in the 60% of patients with baseline LV end-diastolic volume between 200 and 370 mL [36].

**Other cardiovascular settings. **MSCs have also been investigated in dilated cardiomyopathy, refractory angina, peripheral artery disease, and after cardiac surgery [30]. The available evidence base in these settings is smaller and even more heterogeneous, and definitive conclusions are not yet possible.

The contrast between AMI and chronic HF illustrates a broader point. A transient modulatory therapy is most likely to produce a detectable signal when the substrate is itself dynamic. As the disease becomes more structurally fixed, the same biological intervention has less to act upon. This is not a property of MSC therapy alone; it follows from the interaction of any short-lived modulatory signal with disease biology.

## 8. Clinical Evidence and Endpoint Interpretation

The clinical literature on MSC therapy in cardiovascular disease provides several relatively consistent findings and several persistently inconsistent ones. The pattern is informative. Representative clinical studies and meta-analyses of MSC therapy in cardiovascular disease are summarized in Table 1 (Ref. [11,22,23,24,25,26,27,36]).

**Table 1.** **Representative clinical studies and meta-analyses of MSC therapy in cardiovascular disease**.

Study (year)	Setting	Population	n	Cell source/type	Route	Follow-up	Main endpoints	Findings and interpretation
Hare et al., POSEIDON (2012) [24]	ICM	Chronic LV dysfunction due to ICM	30	Auto vs allo BM-MSC (20, 100, 200 × 10^6^ cells)	Transendocardial	13 mo	Safety; LV remodelling; functional capacity	Both arms safe; no significant alloimmune reactions; early enhancement defect reduced ~33%; lowest dose (20 × 10^6^) produced greatest LV-volume reductions and LVEF gain
Heldman et al., TAC-HFT (2014) [25]	Chronic ICM	ICM with LVEF <50%	65 randomized; 59 treated (MSC 19 / BM-MNC 19 / placebo 21)	Autologous BM-MSC vs BM-MNC vs placebo	Transendocardial	12 mo	Safety; MLHFQ; 6MWD; infarct size; LVEF	MSC reduced infarct size −18.9% of LV mass (*p* = 0.004), improved MLHFQ and 6MWD; LVEF and ventricular volumes unchanged
Mathiasen et al., MSC-HF (2015) [26]	Severe ischaemic HF	NYHA II–III; LVEF <45%	60 (2:1)	Autologous BM-MSC	Intramyocardial	6 mo	Primary: LVESV	LVESV −7.6 mL (MSC) vs +5.4 mL (placebo); difference 13.0 mL (*p* = 0.001); LVEF +6.2 points vs placebo (*p* < 0.0001)
Florea et al., TRIDENT (2017) [27]	Chronic ICM	Chronic ICM	30 (15 / 15)	Allogeneic BM-MSC; 20 vs 100 × 10^6^ cells	Transendocardial	12 mo	Safety; LVEF; scar size	Scar size reduced similarly in both groups; LVEF improved only with 100 × 10^6^ dose (+3.7 points, *p* = 0.04)
Bartunek et al., CHART-1 (2017) [36]	Symptomatic ischaemic HF	NYHA II–III/IV; reduced LVEF	271 treated (315 randomised)	MSC-derived cardiopoietic cells	Endomyocardial (retention catheter)	39 wk	Hierarchical composite	Primary endpoint neutral overall (*p* = 0.27); exploratory benefit with LVEDV 200–370 mL (60% of cohort; *p* = 0.015)
Attar et al. (2021) [22]	AMI meta-analysis	Pooled RCTs in AMI	956 (468 / 488) from 13 RCTs	Mixed MSC sources	Mixed (TE, IC)	Variable	LVEF; clinical events	LVEF +3.78% (95% CI 2.14–5.42); first-week +5.74%; effects on clinical events not consistently shown
Lalu et al., SafeCell Heart (2018) [11]	Meta-analysis (AMI + IHF)	23 RCTs (11 AMI + 12 IHF)	23 trials	Adult stem cells incl. MSC	Mixed	Variable	Safety; mortality; LVEF	No acute adverse-event association; LVEF SMD 0.73 (95% CI 0.24–1.21); mortality Peto OR 0.68 (NS)
Jeong et al. (2018) [23]	Meta-analysis	Ischaemic heart disease	950 patients (14 RCTs)	MSC (various)	Mixed	Up to 24 mo	LVEF; scar mass; WMSI	LVEF +3.84% (95% CI 2.32–5.35); reduced scar mass and WMSI; non-significant trends on mortality/rehospitalisation

Abbreviations: 6MWD, six-minute walking distance; BM-MNC, bone marrow mononuclear cells; BM-MSC, bone marrow–derived mesenchymal stromal cells; CMP, cardiomyopathy; HF, heart failure; IC, intracoronary; ICM, ischaemic cardiomyopathy; IHF, ischaemic heart failure; LV, left ventricle; LVEDV/LVESV, left ventricular end-diastolic/end-systolic volume; LVEF, left ventricular ejection fraction; MLHFQ, Minnesota Living With Heart Failure Questionnaire; NYHA, New York Heart Association; RCT, randomised controlled trial; SMD, standardised mean difference; TE, transendocardial; WMSI, wall motion score index; AMI, acute myocardial infarction. Numerical values are taken from the primary publications.

**Feasibility and safety. **Across phase I/II trials of autologous and allogeneic MSCs, with intracoronary, intramyocardial, transendocardial, and intravenous delivery, the procedural and immediate post-procedural safety profile has been broadly acceptable [2,9,24,25,26,27]. The SafeCell Heart systematic review by Lalu and colleagues [11], which pooled 23 trials (11 in AMI, 12 in ischaemic HF), found no association between MSCs and acute adverse events; mortality showed a non-significant trend in favour of MSCs (Peto OR 0.68; 95% CI 0.38–1.22), and overall LVEF improved (standardised mean difference 0.73; 95% CI 0.24–1.21) [11]. Allogeneic preparations have not been associated with major immune-related adverse events at the rates that would limit clinical development; in POSEIDON, allogeneic MSCs did not stimulate significant donor-specific alloimmune reactions [7,24,37].

**Surrogate endpoints. **Modest improvements in LVEF, scar volume, and ventricular volumes have been reported in several trials and pooled analyses. The Jeong meta-analysis (14 randomised controlled trials (RCTs), 950 patients) reported an LVEF increase of 3.84% (95% CI 2.32–5.35) maintained up to 24 months, together with reductions in scar mass and wall motion score index at 6 months [23]. Effect sizes for LVEF are typically of the order of a few percentage points relative to control, with substantial heterogeneity across studies. Some studies have also reported improvements in functional capacity (6-minute walking distance) or quality of life, but findings are not uniform [23,25,36].

**Hard clinical outcomes. **Effects on mortality, hospitalisation, and major cardiovascular events have been more difficult to establish. Most individual trials are not powered to detect small differences in these endpoints, and pooled estimates have generally not shown statistically significant benefit [11,22,23]. The relationship between LVEF improvement and hard outcomes is itself imperfect, which complicates inference from surrogate measures alone.

**Reporting limitations. **A recurring problem is the inconsistent reporting of product characteristics, including viability at infusion, secretory profile, potency assays, and detailed manufacturing methods [31]. As a result, meta-analyses pool studies that may differ substantially at the level of the administered product, and apparent inconsistency may partly reflect this.

Feasibility, safety, biological activity, surrogate endpoint improvement, and durable clinical benefit are not synonymous. Demonstration of one does not entail the others. MSC therapy currently has reasonably strong evidence for the first three. Evidence for surrogate endpoint improvement is real but modest. Evidence for durable clinical benefit remains incomplete. This pattern is compatible both with a genuinely small therapeutic effect and with a larger biological effect that is partly missed by the measurement framework.

## 9. Mechanism–Endpoint Mismatch

**Fig. 1.**
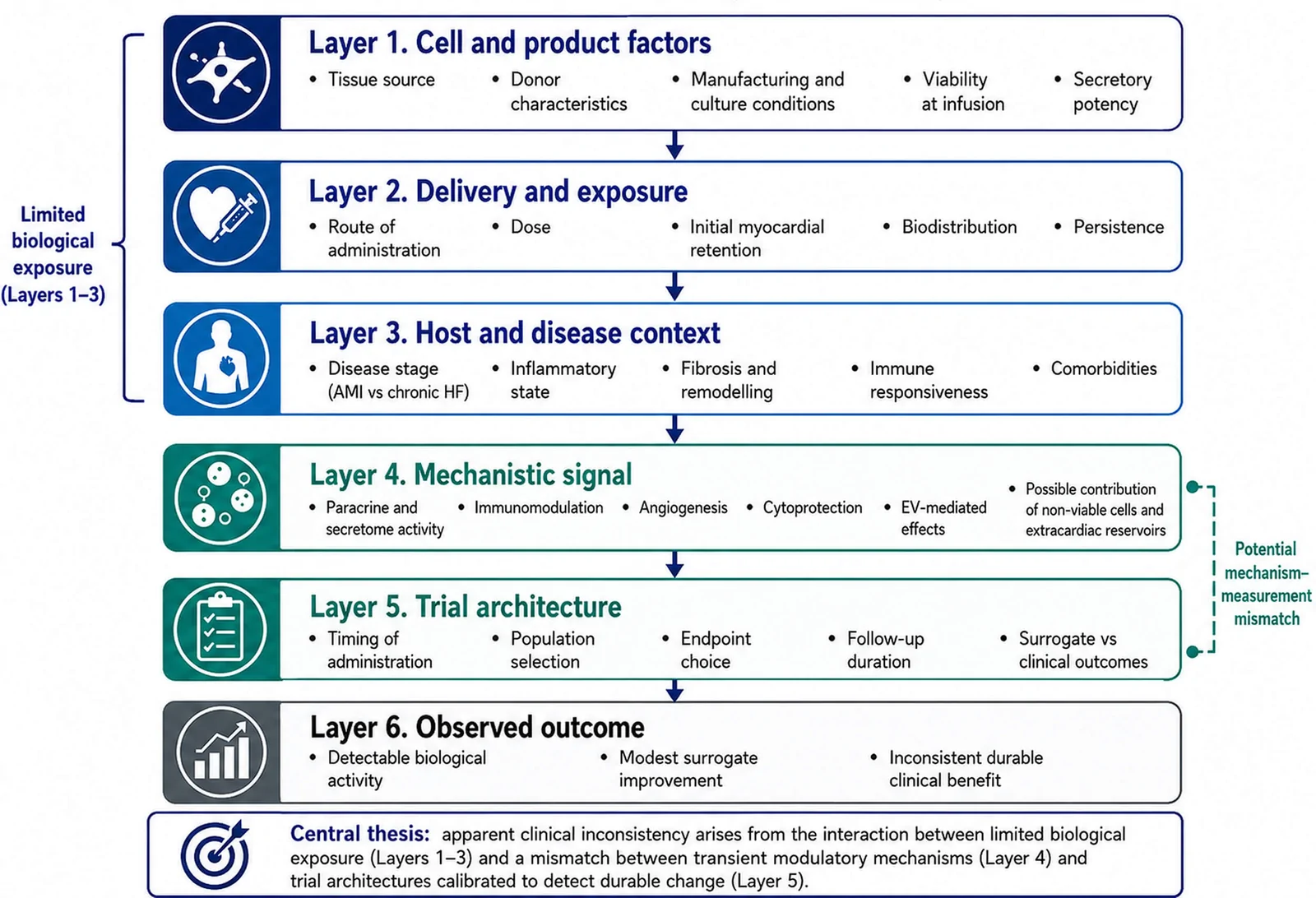
**Conceptual model of clinical inconsistency in MSC therapy for cardiovascular disease**. Schematic representation of the levels at which the translational performance of MSC therapy is determined, organised in six layers connected by directed arrows. Layer 1 (Cell and product factors): tissue source, donor characteristics, manufacturing and culture conditions, viability at infusion, secretory potency. Layer 2 (Delivery and exposure): route of administration, dose, initial myocardial retention, biodistribution, persistence. Layer 3 (Host and disease context): disease stage (AMI versus chronic HF), inflammatory state, fibrosis and remodelling, immune responsiveness, comorbidities. Layer 4 (Mechanistic signal): paracrine and secretome activity, immunomodulation, angiogenesis, cytoprotection, EV-mediated effects, and possible contributions from non-viable cells and extracardiac reservoirs. Layer 5 (Trial architecture): timing of administration, population selection, endpoint choice, follow-up duration, surrogate versus clinical outcomes. Layer 6 (Observed outcome): detectable biological activity, modest surrogate improvement, inconsistent durable clinical benefit. The figure supports the central thesis that apparent clinical inconsistency arises from the interaction between limited biological exposure (layers 1–3) and the structural mismatch between transient modulatory mechanisms (layer 4) and trial architectures calibrated to detect durable change (layer 5).

The central proposition of this review is that part of the clinical inconsistency of MSC therapy may reflect a structural mismatch between the biology of MSCs and the way that biology is measured in cardiovascular trials. Several aspects of this mismatch are worth examining in turn. Fig. 1 summarises how factors at the level of product, delivery, host, mechanism, and trial architecture converge on the observed clinical outcome.

**Temporal scale. **MSC paracrine and immunomodulatory activity is short-lived, constrained by retention and persistence. Cardiovascular endpoints such as LVEF at 6 or 12 months, scar size on late-gadolinium-enhancement magnetic resonance imaging (MRI), end-systolic volume, mortality, and rehospitalisation evolve over time scales an order of magnitude longer than the period of significant cellular activity. A transient signal can plausibly influence early inflammation, microvascular recovery, and the early phase of remodelling without producing a comparably durable change in late structural variables.

**Type of biological effect. **Endpoints derived from a regenerative paradigm—new contractile tissue, increased viable myocardial mass, improved ejection fraction through structural recovery—assume that the therapy adds something durable to the heart. A modulatory therapy does not directly add tissue; it modifies the trajectory of repair. The biological readout is more likely to be subtle: changes in inflammatory biomarkers, microvascular function, edema kinetics, remodelling rate, and immune cell composition. These are seldom primary endpoints in cardiovascular MSC trials.

**Population heterogeneity. **Trials have typically enrolled relatively broad populations, defined by clinical category (ST-elevation myocardial infarction, ischaemic cardiomyopathy with reduced LVEF, refractory angina) rather than by biological responsiveness. The pre-specified subgroup signal in CHART-1, where benefit was suggested only in patients with baseline LV end-diastolic volume between 200 and 370 mL, illustrates how a biologically defined sub-population can show a response that the overall trial does not [36]. A real but conditional effect can therefore appear as a small average effect with wide variance.

**Surrogate-to-clinical translation. **Improvements in LVEF or scar size do not translate linearly into reductions in mortality or hospitalisation. The clinical meaning of a 2–4 percentage point gain in LVEF in a heterogeneous population is itself uncertain. Reliance on surrogates can therefore over- or underestimate the clinical importance of biological activity, depending on the underlying disease and the time horizon considered.

**Endpoints potentially better aligned with MSC biology. **If MSC activity is transient and modulatory, several categories of measurement may be more sensitive than the traditional ones: early inflammatory biomarkers; cardiac MRI parameters of edema, microvascular obstruction, and salvaged myocardium in AMI; quantitative perfusion imaging; rate of change of ventricular volumes rather than absolute values at a fixed timepoint; functional capacity measures; quality of life; and immune phenotyping of circulating cells. Hard clinical outcomes remain relevant but require adequately powered, mechanistically targeted trials to detect realistic effect sizes. Composite endpoints can be useful if their components are aligned with the mechanism rather than aggregated for statistical convenience.

None of this implies that MSC therapy is more effective than current trials suggest. It implies that the current evidence is less informative about the underlying biology than is sometimes assumed. The mismatch hypothesis is testable. Trials designed around the expected temporal and biological signature of MSC activity—appropriate populations, timing, and endpoints—could distinguish a small genuine effect from a measurement artefact. Until such trials are conducted, the persistent gap between biological activity and clinical performance should be interpreted with this caveat in mind.

## 10. Strategies to Improve Effective Biological Exposure

Strategies under investigation to increase the effective biological exposure of the myocardium fall broadly into four groups.

**Biomaterial- and scaffold-based delivery. **Injectable hydrogels, decellularised matrices, and engineered cardiac patches can prolong local cell residence, reduce mechanical loss after intramyocardial injection, and provide a more permissive niche for transplanted cells. Cell sheets and seeded epicardial patches have been explored in chronic ischaemic cardiomyopathy with the aim of improving retention and graft survival [17]. Comparative analyses across delivery routes have argued that bioengineering approaches addressing low retention are likely to be a necessary component of any next-generation cardiac cell therapy [17]. Clinical translation remains early, and most evidence is preclinical.

**Cell engineering and preconditioning. **Hypoxic preconditioning, pharmacological conditioning, and genetic modification (for example, to upregulate prosurvival factors or chemokine receptors) have been explored to improve MSC survival after delivery and to enhance migration to injured tissue [6,13]. The translational pathway is constrained by the additional regulatory complexity of modified cell products.

**Cell-free and secretome-based approaches. **If much of the biological effect is mediated by secreted factors and extracellular vesicles, products derived from MSC conditioned medium or purified EVs may capture part of the activity in a more standardisable form [6,19,20]. Cell-free preparations avoid some of the variability and logistical constraints of live-cell therapy. They introduce new challenges in dose definition, characterisation, and regulatory categorisation.

**Repeated dosing and timing. **Single-dose paradigms may be poorly matched to a transient, short-lived intervention. Repeated administration could, in principle, extend the cumulative biological window, although evidence in cardiovascular trials remains limited. Better matching of delivery timing to disease biology—early after AMI, during periods of active remodelling—may also enhance the probability of detecting a signal, as suggested by the more pronounced LVEF gain observed within the first week post-AMI in pooled analyses [16,22].

These approaches share an underlying assumption: that the rate-limiting step in current MSC therapy is not the biological capacity of the cells but the exposure of the myocardium to that capacity. Whether this assumption is correct will depend on how the field redesigns its trials and on whether improved exposure translates into improved measurable benefit.

## 11. Future Directions

The main translational constraints limiting MSC efficacy and possible mitigation strategies are summarized in Table 2 (Ref. [3,4,11,13,14,17,22,23,24,28,31,34,36]). Several practical recommendations follow from the preceding discussion. They are not exhaustive.

**Table 2.** **Main translational constraints limiting MSC efficacy**.

Constraint	Biological level affected	Mechanistic consequence	Relative importance	Supporting evidence	Possible mitigation
Low myocardial retention	Delivery/exposure	Reduced cumulative paracrine and immunomodulatory dose at the target tissue	High	Biodistribution and labelled-cell tracking [13,17]	Intramyocardial routes; biomaterials; hydrogels; patches
Short cellular persistence	Delivery/exposure	Limits duration of secretory activity; effect confined to early phase	High	BLI and PET imaging; indirect human data [3,4]	Preconditioning; repeated dosing; EV-based products
Product heterogeneity	Product potency	Variable secretome and immunomodulatory capacity between batches	Moderate–high	Potency studies; meta-analytic variance [14,28,31]	Functional release criteria; potency assays
Donor variability	Product potency (autologous)	Age, comorbidity, and metabolic state alter cell function	Moderate	Donor studies; auto vs allo (POSEIDON) [24]	Allogeneic pre-screened donors
Host responsiveness	Recipient biology	Less responsive substrate yields smaller effect for the same signal	Moderate	Subgroup analyses; AMI vs HF contrast [14,34]	Biology-based patient selection
Disease stage	Recipient biology/mechanism	Dynamic substrates (AMI, early HF) more permissive than fixed ones	High	Meta-analytic timing analyses [22]	Align delivery with biological window
Mechanism–endpoint mismatch	Measurement/trial architecture	Transient modulatory effects poorly captured by long-term structural endpoints	High (conceptual)	Surrogate vs hard-outcome inconsistency [11,22,23,36]	Endpoints aligned to mechanism and timing
Reporting inconsistency	Measurement/comparability	Variable product description limits pooled inference	Moderate	Reviews of trial reporting [31]	Standardised reporting guidelines

BLI, bioluminescence imaging; PET, positron emission tomography; EV, extracellular vesicle.

**Product qualification beyond surface markers. **Routine reporting of viability at infusion, potency assays for secretory function, and standardised characterisation of secretome or EV content would substantially improve cross-trial comparability [14,28,33].

**Patient selection. **Enrolment based on biological state—stage of disease, residual viable myocardium, inflammatory or immune profile—rather than broad clinical category may improve the likelihood of detecting a true biological signal. The CHART-1 LV volume subgroup is one example of how such stratification might be applied prospectively [36].

**Timing of administration. **Aligning the timing of delivery with the expected biological window of disease (early after AMI; during active remodelling in HF) is biologically rational and supported by available meta-analytic evidence [22].

**Endpoint selection. **Endpoints should be chosen to match the expected mechanism. Early biomarkers, sensitive imaging measures of edema, perfusion, and remodelling kinetics, and clinical endpoints adequately powered for realistic effect sizes are all part of this realignment. Surrogate endpoints retain a role in early-phase development; hard outcomes should be tested only when prior phases have established a coherent biological and clinical signal.

**Mechanism-specific trial design. **Treating MSCs as a uniform regenerative intervention has limited interpretability of past trials. Future studies could be designed to test mechanism-specific hypotheses—for example, modulation of early inflammation in AMI, or improvement of microvascular function in chronic ischaemia—rather than generic regenerative benefit [16].

## 12. Conclusions

MSC therapy in cardiovascular disease shows reproducible biological activity, modest and variable improvements in surrogate endpoints, and no consistent demonstration of durable clinical benefit. Reduced myocardial exposure, product and donor variability, and host heterogeneity are real and important constraints. They are unlikely to account, on their own, for the entirety of the observed inconsistency.

Part of the translational difficulty may reflect a mismatch between transient, modulatory, context-dependent MSC biology and trial architectures calibrated to detect durable structural or hard clinical change. A short-lived modulatory signal may generate effects that are biologically meaningful but inadequately captured by current endpoints.

MSC therapy in cardiovascular disease may be better understood as a transient, context-dependent biological intervention rather than as a durable regenerative replacement strategy. Future development is likely to require closer alignment between product definition, delivery, patient selection, timing, and endpoints sensitive to the biological window in which MSCs are most likely to act.
